# Cell Population Data (CPD) for Early Recognition of Sepsis and Septic Shock in Children: A Pilot Study

**DOI:** 10.3389/fped.2021.642377

**Published:** 2021-03-08

**Authors:** Paolo Biban, Martina Teggi, Marcella Gaffuri, Pierantonio Santuz, Diletta Onorato, Giovanni Carpenè, Dario Gregori, Giuseppe Lippi

**Affiliations:** ^1^Pediatric Intensive Care Unit, Division of Pediatric Critical and Emergency Care, Verona University Hospital, Verona, Italy; ^2^Section of Clinical Biochemistry, University of Verona, Verona, Italy; ^3^Unit of Biostatistics, Epidemiology and Public Health, Department of Cardiac, Thoracic, Vascular Sciences and Public Health, University Hospital of Padua, Padova, Italy

**Keywords:** cell population data, sepsis, septic shock, diagnosis, prognosis, child

## Abstract

**Objectives:** Innovative Cell Population Data (CPD) have been used as early biomarkers for diagnosing sepsis in adults. We assessed the usefulness of CPD in pediatric patients with sepsis/septic shock, in terms of early recognition and outcome prediction. We revised 54 patients (0–15 y) admitted to our Pediatric Intensive Care Unit (PICU) for sepsis/septic shock during a 4-year period. Twenty-eight patients were excluded, 26 septic patients were enrolled (G1). Forty children admitted for elective surgery served as controls (G2). Data on five selected CPD parameters, namely neutrophils fluorescence intensity (NE-SFL), monocytes cells complexity (MO-X), monocytes fluorescence intensity (MO-Y), monocytes complexity and width of dispersion of events measured (MO-WX), and monocytes cells size and width dispersion (MO-WZ), were obtained at time of PICU admission (*t0*) by a hematological analyzer (Sysmex XN 9000®). As the primary outcome we evaluated the relevance of CPD for diagnosing sepsis/septic shock on PICU admission. Furthermore, we investigated if CPD at *t0* were correlated with C-reactive protein (CRP), patient survival, or complicated sepsis course.

**Results:** On PICU admission (*t0*), NE-SFL, MO-WX, and MO-Y were higher in sepsis/septic shock patients compared to controls. NE-SFL values were correlated with CRP values in G1 patients (*r* = 0.83). None of the five CPD parameters was correlated with survival or complicated sepsis course.

**Conclusion:** We found higher values of NE-SFL, MO-WX, and MO-Y in children with sepsis/septic shock upon PICU admission. These parameters may be a promising adjunct for early sepsis diagnosis in pediatric populations. Larger, prospective studies are needed to confirm our preliminary observations.

## Introduction

Sepsis is a major cause of morbidity and mortality, accounting for more than 30 million cases each year worldwide and 6 million deaths (1–3). Globally, about 1.2 million cases of childhood sepsis are estimated each year, with mortality rates largely varying from 4 to 50%, depending on illness severity, risk factors, and geographic location (4). In 2005, a definition of sepsis in children was proposed by Goldstein et al. (5). However, specific criteria used for identifying children with sepsis have not been rigorously evaluated (4–7). Recently, a Pediatric Sepsis Definition Taskforce has synthesized all available evidence for identifying children who have or may develop sepsis-associated organ dysfunction, as well as those with sepsis who may be at higher risk of progressing to multiple organ dysfunction (MOD) or death (8).

Early diagnosis, combined with an appropriate management in the first hour of hospital admission (“The Hour-1 Bundle”), remains crucial for patients with the most severe infections, ideally before any signs and symptoms of organ failure have appeared (9). However, the timely diagnosis of sepsis remains an ongoing challenge for any clinician (10–12). Currently, diagnosis of sepsis is based upon clinical parameters and laboratory findings. Blood culture is the gold standard technique for identifying pathogenic microorganisms, yet it has drawbacks, including long turnaround time, low sensitivity, large volume of blood required, risk of false negative results after initiation of antibiotic therapy, and vulnerability to pre-analytical variables (13, 14). White blood cell (WBC) count is regularly obtained in sepsis workup, but often it has unreliable specificity for detecting sepsis in several populations (15). Thus, a vast array of serum (or plasma) sepsis biomarkers have been tested over the past decades, both in adult and pediatric populations (13, 16–18). These typically include C-reactive protein (CRP), procalcitonin (PCT), presepsin, interleukin 6 (IL-6), and neutrophil CD64, among others (19–23). At present, PCT and presepsin appear to be the most promising tests for early diagnosis of sepsis, for prognostic information, and therapeutic decision-making (24, 25). Nonetheless, these two biomarkers provide suboptimal diagnostic accuracy and do not offer information on causative microorganism(s) (13, 18, 25).

Thus, the focus on early sepsis detection has fueled interest in the identification of new, low-cost, routinely available biomarkers, capable of discriminating, early in the process, between patients with and without infection. Ideally, these biomarkers should improve sepsis recognition and management through three main applications: diagnosis, monitoring response to treatment, and risk assessment/stratification (24, 26).

Recently, few studies indicated that cell population data (CPD) might offer interesting quantitative information on morphological and functional characteristics of leukocytes (neutrophils, monocytes, and lymphocytes). The variation of CPD in response to various stimuli (e.g., infections) was found to provide rapid information on leucocytes activation, offering a new method for improving early diagnosis of sepsis (27–31). Notably, CPD parameters can be automatically obtained by a new generation of hematological analyzers (e.g., Sysmex XN) during standard differential cell blood count analysis, obviating the need for additional blood tests and added costs (31–36). The CPD analysis method is therefore characterized by simplicity, accessibility, and speed.

The present study aimed to assess the clinical relevance of CPD as parameters for early diagnosis of sepsis or septic shock in pediatric patients, as well as to test CPD for outcome prediction.

## Methods

This was a retrospective, observational, single-center case-control study, conducted in the Department of Neonatal and Pediatric Critical Care, Verona University Hospital (Italy).

Patients' demographics and clinical characteristics were obtained from our hospital health records database.

We considered all critically ill children admitted to PICU for clinically suspected or confirmed diagnosis of sepsis or septic shock, between March 2016 and March 2020.

### Inclusion Criteria

Patients were eligible (study group—G1) if they met the following criteria: (a) confirmed sepsis or septic shock diagnosis according to the 2005 definitions (5); (b) PICU admission at sepsis or septic shock onset *(t0)*; (c) complete blood count and CRP value obtained on PICU admission; (d) age: 0–15 years.

Additionally, we divided the G1 study group into two subgroups (G1a and G1b), based on patients' uncomplicated or complicated sepsis course during their PICU stay, respectively. Patients with a complicated sepsis course (G1b), either had severe sepsis or died from sepsis-induced multiple organ failure, as defined by Goldstein et al. (5).

### Exclusion Criteria

(a) unconfirmed diagnosis of sepsis or septic shock; (b) missing CPD parameters; (c) underlying hemato-oncological disease; (d) age > 15 years.

### Control Group

A convenient group of children aged 0–15 years, without signs of infection, extracted from those admitted in the PICU and undergoing routine blood testing before elective surgery, served as control group (control group—G2).

### Cell Population Data

CPD were obtained with an automated hematological analyzer (Sysmex XN 9000®, Sysmex Corporation, Kobe, Japan), which uses flow cytometry to measure individual cells. Activation of neutrophils and monocytes are detected in real-time, in an accurate and reproducible way (35). In the Sysmex XN analyzers, the leukocyte differential channel discriminates the leukocytes, while the signals are plotted in a scattergram. In brief, the differential leucocyte count is based on criteria of cellular granularity (side scatter light), cell volume and shape (forward scatter light) and nucleic acid/protein content of cells (fluorescent light intensity), by preincubation with unique surfactant reagents and fluorescence staining (30, 35). These optical signals of leukocyte differential are presented in the three axes of the white blood cells differential fluorescence (WDF) channel scattergram.

Among the whole panel of CPD measured by Sysmex XN 9000®, we selected *a priori* five specific parameters: neutrophils fluorescence intensity (NE-SFL), monocytes cells complexity (MO-X), monocytes fluorescence intensity (MO-Y), monocytes complexity and width of dispersion of the events measured (MO-WX), and monocytes cells size and width dispersion (MO-WZ). CPD and morphological and functional characteristics of leukocytes are summarized in Supplementary Table 1.

### Sample Collection

In the study group (G1), blood samples were collected on PICU admission (*t0*), at onset of sepsis/septic shock. In the control group (G2), blood samples were obtained for routine pre-surgery testing.

We retrieved data on selected CPD of neutrophils and monocytes, from the blood sample database of our central medical laboratory (laboratory information system; LIS).

### Primary Outcome

We evaluated if median values of five selected CPD (NE-SFL, MO-X, MO-Y, MO-WX, MO-WZ) collected in septic children at *t0* (on PICU admission) were different from those of control group.

### Secondary Outcomes

We investigated if CPD parameters at *t0* correlated with CRP, as well as with patient survival and severity of clinical course. We also compared CPD collected at *t0* in survivors vs. non-survivors, and in patients with complicated vs. uncomplicated course.

### Ethics

Informed consent and ethical committee approval were waived as the analysis used anonymous clinical data and the design of the study was retrospective. Privacy Officer of the Verona University Hospital authorized personal data processing and analysis.

### Statistical Analysis

Statistical analysis was performed using the R 3.6.2 system. The Kolmogorov-Smirnov test was used to determine the normality of distribution of continuous variables, with *p* > 0.05 considered as non-significant. The data description procedure has been performed reporting continuous data as median (I, III quartiles). Differences between the two groups were assessed using Student's *t*-test for normally and Wilcoxon-type tests for non-normally distributed variables. The Pearson chi-square test, or Fisher-exact test (when appropriate) were used for categorical variables. An Ordinary Least Square model has been computed to evaluate the covariate effect on PICU length of stay. Pearson's correlation analysis was also performed between CPD and CRP.

## Results

### Population

A total of 54 eligible patients were screened for the present study. Twenty-eight patients (51.8%) were excluded: 16 due to missing CPD (29.6%), six for uncertain sepsis diagnosis (11.1%) and six for underlying hemato-oncological disorders (11.1%). Therefore, 26 out of 54 patients (48.2%) were finally enrolled in the study (G1). Main clinical characteristics are shown in Table 1. Thirteen patients were males (50%). Median age was 2 months (range 0–13 years). Seven patients had <28 days of life (neonates). Clinical diagnosis upon PICU admission was sepsis in 20 patients (76.9%) and septic shock in 6 (23.1%). WBC count, hemoglobin and platelets were not different between sepsis and septic shock patients (*p*> 0.05). The most common source of sepsis/septic shock was primary bloodstream infection (50%). At least one microorganism was isolated in 21 patients (80.8%), with predominant organisms being Gram-negative bacteria. Twenty-three patients survived (88.5%), whilst three eventually died (11.5%). Ten patients out of 26 had co-morbidities (38.5%), seven of whom in survivors (30.4%) and three in non-survivors (100%). In this latter subset, two had complex congenital heart diseases and one myocarditis. The cause of death was sepsis-induced multiple organ failure (Table 1).

**Table 1:** Demographics and clinical characteristic of 26 sepsis/septic shock patients (G1 group) and control group (G2).

**Variables**	**All patients (G1)**	**Sepsis**	**Septic shock**	**Control group (G2)**
Patients, No (%)	26	20 (76.9%)	6 (23.1%)	40
Age (months) mean/median (IQR)	26.5/2 (1–33)	24/2 (1–36)	27/2.5 (1–18)	78/60 (36–120)
Female, No (%)	13 (50)	10 (50)	3 (50)	9 (22.5)
Male, No (%)	13 (50)	10 (50)	3 (50)	31 (77.5)
**ADMISSION SOURCE, NO (%)**				
Emergency Department	10 (38.5)	8 (40)	2 (33.4)	
Cardiothoracic ICU	3 (11.5)	3 (15)	0	
Pediatric Department	5 (19.3)	4 (20)	1 (16.6)	
Others Hospitals	6 (23)	3 (15)	3 (50)	
Neonatal Intensive Care Unit	2 (7.7)	2 (10)	0	
**COMORBIDITIES, NO (%)**				
Complex congenital heart disease	8 (30.7)	7 (35)	1 (16.6)	
Polymalformative Syndrome	3 (11.5)	3 (15)	0	
**BLOOD COUNT, MEDIAN (IQR)**				
Hemoglobin (g/dl)	10.7 (9.5–11.8)	10.9 (9.5–12)	10.5 (10.1–11.2)	
Platelets (10^∧^9/L)	263.5 (172.5–376.5)	267 (210–392)	148.5 (53–325)	
White Blood Cells (10^∧^9/L)	10.3 (7.1–17.3)	12.8 (7.4–18.5)	9.6 (4.7–10.7)	
Neutrophil (10^∧^9/L)	5.6 (2.6–8)	7.4 (2.9–9.3)	1.4 (1.2–4)	
CRP mg/L, median (IQR)	69 (13.3–170.5)	69 (15.7–164.7)	44.5 (9.5–222.7)	
**SOURCE OF INFECTION, NO (%)**				
Bloodstream infection	13 (50)	10 (50)	3 (50)	
Neurological infection	4 (15.4)	1 (5)	3 (50)	
Respiratory infection	5 (19.2)	5 (25)	0	
Abdominal infection	2 (7.7)	2 (10)	0	
Urinary tract infection	2 (7.7)	2 (10)	0	
**MICROBIOLOGICAL ANALYSIS RESULTS, NO (%)**				
Gram-positive	7 (26.9)	4 (20)	3 (50)	
Gram-negative	9 (34.7)	6 (30)	3 (50)	
Virus	4 (15.4)	4 (20)	0	
Fungi	1 (3.8)	1 (5)	0	
No organism identified	5 (19.2)	5 (25)	0	
Continuous renal replacement therapy, No (%)	1 (3.8)	1 (5)	0	
Extracorporeal Membrane Oxygenation, No (%)	1 (3.8)	1 (5)	0	
**MECHANICAL VENTILATIONS, NO (%)**				
Invasive	10 (38.4)	6 (23)	4 (66.6)	
Non Invasive	15 (57.7)	10 (50)	5 (83.3)	
**VENTILATOR DAYS, MEDIAN (IQR)**				
Invasive	0 (0–6.7)	0 (0–1.5)	6.5 (1.5–13.7)	
Non Invasive	2 (0–5.7)	0.5 (0–6)	2.5 (2–3.7)	
Required vasoactive infusion, No (%)	9 (34.6)	5 (19.2)	4 (66.6)	
Vasoactive Infusions, days, median (IQR)	0 (0–2)	0 (0–0.2)	2 (0.5–2)	
PICU LOS, days, median (IQR)	12.5 (3–26.5)	9.5 (1.7–18.5)	27.5 (17.2–42.2)	
Hospital LOS, days, median (IQR)	22 (14–42.7)	17.5 (10.7–34.5)	44.5 (30.7–48.5)	
PICU outcome, No (%)				
*Discharge*	23 (88.5)	17 (85)	6 (100)	
*Death*	3 (11.5)	3 (15)	0	

*CRP, C-reactive protein; PICU, pediatric intensive care unit; LOS, length of stay*.

Forty patients, (31 males, 9 females), with a median age of 5 years (IQR 3–10 years), were enrolled in the control group (G2).

### CPD Upon PICU Admission *(t0)* in Sepsis/Septic Shock Patients (G1) and in Controls (G2)

At the time of PICU admission (*t0*), three CPD parameters, namely NE-SFL, MO-WX, and MO-Y, were significantly higher in children with sepsis/septic shock (G1) compared to controls (G2): [(NE-SFL 52.5 vs. 46.8; *p* < 0.0001); (MO-WX 272 vs. 249.5; *p*=0.004); (MO-Y 111.5 vs. 108.1; *p*=0.04)] (Table 2). Similarly, MO-X values were significantly higher, but only in septic shock patients compared to controls (Supplementary Table 2). MO-WZ median values were not different between G1 and G2 patients (*p* = 0.64). In terms of accuracy, AUC (95% CI) was 0.84 (0.71–0.97) for NE-SFL (*p* < 0.0001), 0.69 (0.56–0.83) for MO-WX (*p* < 0.05), and 0.61 (0.45–0.67) for MO-Y (*p*=0.089).

**Table 2:** CPD values upon PICU admission *(t0*) in patients with sepsis/septic shock (G1 group) and controls (G2).

**CPD at *t0* median (IQR)**	**G1—Sepsis/septic shock group** **(*n* = 26)**	**G2—Control group** **(*n*=40)**	***P-value***
NE-SFL	52.5 (50.0–58.9)	46.8 (45.9–47.8)	<0.0001
MO-X	122.2 (117.7–125)	121 (119.7–122.1)	0.15
MO-Y	111.5 (105.1–122.9)	108.1 (102.8–111.3)	0.04
MO-WX	272 (249.5–300.7)	249.5 (231–269)	0.004
MO-WZ	587 (525–598)	572.5 (526.5–629.5)	0.64

*Data expressed as median and interquartile range (IQR). CPD, cell population data*.

In a subgroup analysis of G1, we compared median (IQR) CPD values of seven neonates with those of 19 older patients: NE-SFL were 56.3 (42.2–65.5) vs. 52.2 (50–59.3), respectively (*p*=0.47). MO-WX were 352 (278–360) vs. 261 (237–289) (*p* < 0.001), while MO-Y were 94.3 (88.7–111.4) vs. 116.4 (106.4–132.3) (*p* < 0.01), respectively.

### CPD Upon PICU Admission (*t0*) and Correlation With CRP, Survival, and Complicated Sepsis Course

Overall, in the 26 G1-patients a good correlation was found between NE-SFL and CRP values at *t0* (*r* = 0.83) (Supplementary Figure 1). Such correlation was even stronger when calculated in the 13 G1-patients with positive blood-culture (*r*=0.88).

Conversely, even though NE-SFL and MO-WX were slightly higher in three non-survivors compared to 23 survivors [NE-FSL = 59.3 (55.1–66.9 IQR) vs. 52.2 (49.4–57.7 IQR), respectively; *p*=0.24]; [MO-WX = 332 (294.5–342 IQR) vs. 271 (246–295 IQR), respectively; *p*=0.19], none of the five CPD parameters measured at *t0* was significantly correlated with survival.

Ten patients (38.5%) had complicated sepsis courses and displayed significantly longer PICU-LOS than 16 patients with uncomplicated sepsis courses (*p* < 0.001) (data not shown). Their CPD values were not statistically different from those patients with uncomplicated courses (Supplementary Table 3).

The predictive univariate models included several parameters at *t0*, namely NE-SFL, MO-X, MO-Y, MO-WX, MO-WZ, WBC counts, hemoglobin, and platelets. None of these variables were associated with PICU-LOS.

## Discussion

The main findings of our study were that three parameters of CPD, namely NE-SFL, MO-WX, and MO-Y, displayed significantly higher values in children with sepsis or septic shock on PICU admission compared to controls. We also observed a significant correlation between NE-SFL and CRP values at *t0*, especially in septic patients with positive blood-culture. Our preliminary results suggest that selected CPD parameters may aid clinicians in confirming the diagnosis of sepsis early in the course of severe infections in children.

Sepsis still represents one of the prominent causes of morbidity and mortality in critically ill patients of any age (1, 37). Early diagnosis and treatment are essential to improve outcomes, mainly by correcting shock and organ dysfunction before the occurrence of irreversible damage. According to the 2016 Sepsis-3 definition, sepsis is a life-threatening organ dysfunction caused by a dysregulated host response to infections, where an exaggerated immune response may result in collateral damage and death of host cells and tissues (38). The early step in initiation of host response to pathogens encompasses the activation of innate immune system, primarily entailing neutrophils and monocytes (39). A new generation of hematological analyzers has recently enabled clinicians to obtain faster and even more accurate morphological information from the complete blood count (34). In particular, leukocyte-derived parameters, named Cell Population Data, are emerging as potentially useful parameters for early sepsis diagnosis in adult patients (35).

The primary outcome of our pilot study was to evaluate the relevance of five parameters of CPD, obtained at PICU admission, in order to confirm the diagnosis of sepsis/septic shock in children. NE-SFL exhibited the best accuracy in discriminating septic patients, with significantly higher NE-SFL values at *t0* in septic children compared to controls. Notably, the subgroup of children with septic shock had higher NE-FSL values than septic children. NE-FSL quantifies neutrophil immaturity and activation. In particular, high fluorescent intensity indicates an increase in RNA and DNA cell content, which reflects large cytokines production. During the early stage of sepsis, transition of neutrophils from a resting state within the circulation to activated state at infection site is triggered by an ordered sequence of signals from priming stimuli (e.g., C5a, lipopolysaccharide and cytokines) (40). Thus, consistent with the function of neutrophils as first responders during innate immune response to invading organisms and morphological characteristics of these activated cells, our data indicated that NE-SFL could be useful for early sepsis (and septic shock) detection. Our results are in accordance with previous studies in adulthood. In a recent study on 130 adults with sepsis, Park *et al*. suggested that both NE-SFL and NE-WY could be useful for detecting sepsis, together with other currently used surrogate sepsis biomarkers (30). Buoro et al. reported that NE-SFL and MO-X items exhibited the best CPD diagnostic performance, being found significantly higher in 40 adults with sepsis compared to controls (33). Similarly to our results, NE-SFL values were significantly higher in patients with septic shock compared to those with sepsis (33). In another study, NE-SFL and MO-X proved to be the most relevant CPD parameters in predicting sepsis in a group of 137 adults (31).

In our study, MO-WX values were also significantly increased in septic patients compared to controls, showing a discreet accuracy. This parameter reflects the degree of heterogeneity of monocytes complexity. Monocytes are a heterogeneous set of cells, which differ in phenotype, size, nuclear morphology, and function. In sepsis, this variability is even more pronounced, reflected by a variation in monocytes complexity (width of dispersion) (41). Our results are in agreement with previous reports on early changes in monocytes in adult septic patients (31, 34). In 2019, Shalini et al. observed significantly increased values of five CPD monocytes parameters in 100 adult patients with clinically suspected sepsis (34). Interestingly, they reported NE-SFL, MO-WX, and MO-Y values quite similar to those observed in our study, both in the sepsis and control groups. Even though we may speculate that different ages may not necessarily affect the absolute values of these three CPD parameters, further research is still needed to clarify this issue.

Of note, in a subgroup analysis we observed that CPD values of seven neonates were not consistent with those found in the whole sepsis group. In fact, while NE-SFL levels were comparable, neonates had significantly higher MO-WX and lower MO-Y values compared to older septic patients. However, given the very small number of patients, these findings must be interpreted with caution.

As secondary outcomes, we investigated whether or not CPD may correlate at *t0* with CRP, patient survival, and severity of sepsis course. Our analyses revealed a good correlation between NE-SFL and CRP values at PICU admission, even stronger in patients with positive blood culture.

Differently, CPD parameters at *t0* were not correlated with patient survival and severity of sepsis course. Similarly, Park *et al*. evaluated NE-SFL for discriminating sepsis severity and predicting mortality in adults, with unsatisfactory evidence (30). Conversely, in forty adults with sepsis or septic shock, Buoro *et al*. reported a correlation between NE-SFL and MO-X values with Sequential Organ Failure Assessment (SOFA) score, a validated index for predicting clinical outcomes of critically ill adult patients (33). The authors suggested that NE-SFL and MO-X might provide useful information for diagnosis as well as for management of septic patients in ICU.

At present, data on the role of CPD for diagnosing sepsis in pediatric populations are scant. In 2010, Raimondi et al. investigated mean neutrophil volume (MNeV) and its standard deviation (neutrophil distribution width) as a screening for late-onset neonatal sepsis in 120 preterm infants (42). The combination of MNeV with CRP showed a good diagnostic performance for either suspecting or ruling out late-onset sepsis in their series (42). In 2012, Jung et al. investigated CPD for differential diagnosis of viral infection in 602 children with various conditions (43).

To our knowledge, the present study is one of the first to explore the relevance of leukocytes CPD in diagnosing sepsis in children, by means of a modern automated blood analyzer.

Our study has several limitations. First, it is a single-center, retrospective study with a relatively small sample size, so that results shall be regarded as preliminary. Second, we assumed the onset of sepsis was close to PICU admission, though we could not retrieve accurate data on this assumption. However, all analyzed CPD parameters were collected in the PICU concomitantly with the sepsis onset. Lastly, the study group had a median age significantly lower than controls, potentially introducing a risk of selection bias.

Nonetheless, we believe our pilot study does offer some new information on the potential role of CPD in early detection of sepsis in critically ill children, thus allowing a more timely clinical management of this life-threatening condition.

However, further research on larger populations would be warranted for investigating the relevance of CPD as diagnostic parameters in septic patients. We speculate that CPD may also be useful for guiding treatment during the sepsis course, and for providing prognostic indications.

## Conclusions

In this study, we observed significant increases in NE-SFL, MO-WX, and MO-Y in children with sepsis and septic shock, at the time of PICU admission. Our findings may suggest a role of CPD to serve as quick and low-cost parameters in early detection of sepsis, also in pediatric populations. Larger, prospective trials are warranted to confirm our preliminary results. Furthermore, we speculate that serial CPD measurements could support clinicians in tracking response to therapy and in predicting clinical outcomes in children with sepsis and septic shock.

## Data Availability Statement

The raw data supporting the conclusions of this article will be made available by the authors, without undue reservation, to any qualified researcher.

## Ethics Statement

Ethical review and approval was not required for the study on human participants in accordance with the local legislation and institutional requirements. Written informed consent from the participants' legal guardian/next of kin was not required to participate in this study in accordance with the national legislation and the institutional requirements.

## Author Contributions

PB and MT contributed to conception and design of the study. PS, MT, DO, and GC organized the database. DG performed the statistical analysis. PB and MT wrote the first draft of the manuscript. MG, DO, GC, and GL wrote sections of the manuscript. All authors contributed to manuscript revision, read, and approved the submitted version.

## Conflict of Interest

The authors declare that the research was conducted in the absence of any commercial or financial relationships that could be construed as a potential conflict of interest.

## References

[1] Angus DC Linde-Zwirble WT Lidicker J Clermont G Carcillo J Pinsky MR. Epidemiology of severe sepsis in the United States: analysis of incidence, outcome, and associated costs of care. Crit Care Med. (2001) 29:1303–10. 10.1097/00003246-200107000-00002 11445675

[2] Sehgal M Ladd HJ Totapally B. Trends in epidemiology and microbiology of severe sepsis and septic shock in children. Hosp Pediatr. (2020) 10:1021–30. 10.1542/hpeds.2020-0174 33208389

[3] Gyawali B Ramakrishna K Dhamoon AS. Sepsis: the evolution in definition, pathophysiology, and management. SAGE Open Med. (2019) 7:2050312119835043. 10.1177/2050312119835043 30915218 PMC6429642

[4] Weiss SL Peters MJ Alhazzani W Agus MSD Flori HR Inwald DP. Surviving sepsis campaign international guidelines for the management of septic shock and sepsis-associated organ dysfunction in children. Intensive Care Med. (2020) 46:10–67. 10.1007/s00134-019-05878-6 32030529 PMC7095013

[5] Goldstein B Giroir B Randolph A. International consensus conference on pediatric sepsis. international pediatric sepsis consensus conference: definitions for sepsis and organ dysfunction in pediatrics. Pediatr Crit Care Med. (2005) 6:2–8. 10.1097/01.PCC.0000149131.72248.E6 15636651

[6] Matics TJ Sanchez-Pinto LN adaptation and validation of a pediatric sequential organ failure assessment score and evaluation of the sepsis-3 definitions in critically ill children. JAMA Pediatr. (2017) 171:e172352 20. 10.1001/jamapediatrics.2017.2352 28783810 PMC6583375

[7] Schlapbach LJ Straney L Bellomo R MacLaren G Pilcher D. Prognostic accuracy of age-adapted SOFA, SIRS, PELOD-2, and qSOFA for in-hospital mortality among children with suspected infection admitted to the intensive care unit. Intensive Care Med. (2018) 44:179–88. 10.1007/s00134-017-5021-8 29256116 PMC5816088

[8] Menon K Schlapbach LJ Akech S Argent A Chiotos K Chisti MJ. Pediatric sepsis definition-a systematic review protocol by the pediatric sepsis definition taskforce. Crit Care Explor. (2020) 2:e0123. 10.1097/CCE.0000000000000123 32695992 PMC7314341

[9] Mitchell LM Laura EE Andrew R. The surviving sepsis campaign bundle: 2018 update. Crit Care Med. (2018) 46:997–1000 10.1097/CCM.0000000000003119 29767636

[10] Schlapbach LJ. Paediatric sepsis. Curr Opin Infect Dis. (2019) 32:497–504. 10.1097/QCO.0000000000000583 31335441

[11] Biban P. Pediatric Septic shock in the emergency department: can we set the alarm clock a little forward? Pediatr Crit Care Med. (2016) 17:1011–2. 10.1097/PCC.0000000000000924 27705992

[12] Weiss SL Fitzgerald JC Balamuth F Alpern ER Lavelle J Chilutti M. Delayed antimicrobial therapy increases mortality and organ dysfunction duration in pediatric sepsis. Crit Care Med. (2014) 42:2409–17. 10.1097/CCM.0000000000000509 25148597 PMC4213742

[13] Lippi G. Sepsis biomarkers: past, present and future. Clin Chem Lab Med. (2019) 57. 10.1515/cclm-2018-1347 30710482

[14] Sinha M Jupe J Mack H Coleman TP Lawrence SM Fraley SI. Emerging technologies for molecular diagnosis of sepsis. Clin Microbiol Rev. (2018) 31:e00089–e00017. 10.1128/CMR.00089-17 29490932 PMC5967692

[15] Crouser ED Parrillo JE Seymour C Angus DC Bicking K Tejidor L. Improved early detection of sepsis in the ED with a novel monocyte distribution width biomarker. Chest. (2017) 152:518–26. 10.1016/j.chest.2017.05.039 28625579 PMC6026271

[16] Dolin HH Papadimos TJ Stepkowski S Chen X Pan ZK. A novel combination of biomarkers to herald the onset of sepsis prior to the manifestation of symptoms. Shock. (2018) 49:364–70. 10.1097/SHK.0000000000001010 29016484 PMC5811232

[17] Larsen FF Petersen JA. Novel biomarkers for sepsis: a narrative review. Eur J Intern Med. (2017) 45:46–50. 10.1016/j.ejim.2017.09.030 28965741

[18] Wong HR. Sepsis biomarkers. J Pediatr Intensive Care. (2019) 8:11–6. 10.1055/s-0038-1677537 31073503 PMC6506678

[19] Bartoletti M Antonelli M Bruno Blasi FA Casagranda I Chieregato A Fumagalli R. Procalcitonin-guided antibiotic therapy: an expert consensus. Clin Chem Lab Med. (2018) 56:1223–9. 10.1515/cclm-2018-0259 29874192

[20] Hayashida K Kondo Y Hara Y Aihara M Yamakawa K. Head-to-head comparison of procalcitonin and presepsin for the diagnosis of sepsis in critically ill adult patients: a protocol for a systematic review and meta-analysis. Br Med J Open. (2019) 7:e014305. 10.1136/bmjopen-2016-014305 28264831 PMC5353338

[21] Santuz P Soffiati M Dorizzi RM Benedetti M Zaglia F Biban P. Procalcitonin for the diagnosis of early-onset neonatal sepsis: a multilevel probabilistic approach. Clin Biochem. (2008) 41:1150–5. 10.1016/j.clinbiochem.2008.05.015 18606160

[22] Pugni L Pietrasanta C Milani S Vener C Ronchi A Falbo M. Presepsin [soluble CD14 subtype]: reference ranges of a new sepsis marker in term and preterm neonates. PLoS ONE. (2015) 10:e0146020. 10.1371/journal.pone.0146020 26720209 PMC4697794

[23] Yang HS Hur M Yi A Kim H Lee S Kim SN. Prognostic value of presepsin in adult patients with sepsis: systematic review and meta-analysis. PLoS ONE. (2018) 13:e0191486. 10.1371/journal.pone.0191486 29364941 PMC5783380

[24] Jacobs L Wong HR. Emerging infection and sepsis biomarkers: will they change current therapies? Expert Rev Anti Infect Ther. (2016) 14:929–41. 10.1080/14787210.2016.1222272 27533847 PMC5087989

[25] Endo S Suzuki Y Takahashi G Shozushima T Ishikura H Murai A. Presepsin as a powerful monitoring tool for the prognosis and treatment of sepsis: a multicenter prospective study. J Infect Chemother. (2014) 20:30–4. 10.1016/j.jiac.2013.07.005 24462421

[26] Marshall JC Reinhart K; International Sepsis Forum. Biomarkers of sepsis. Crit Care Med. (2009) 37:2290–8. 10.1097/CCM.0b013e3181a02afc 19487943

[27] Green R. Development, history, and future of automated cell counters. Clin Lab Med. (2015) 35:1–10. 10.1016/j.cll.2014.11.003 25676368

[28] Xu D. Clinical applications of leukocyte morphological parameters. Int J Pathol Clin Res. (2015) 1:1–4. 10.23937/2469-5807/1510002

[29] Hoffmann JJML. New hematology analyzer parameters and their clinical relevance. EFLM Newsl. (2018) 1:4–5.

[30] Park SH Park CJ Lee BR Nam K-S Kim M-J Han M-Y. Sepsis affects most routine and cell population data (CPD) obtained using the Sysmex XN-2000 blood cell analyzer: neutrophil-related CPD NE-SFL and NE-WY provide useful information for detecting sepsis. Int J Lab Hematol. (2015) 37:190–8. 10.1111/ijlh.12261 24867378

[31] Urrechaga E Bóveda O Aguirre U. Role of leucocytes cell population data in the early detection of sepsis. J Clin Pathol. (2018) 71:259–66. 10.1136/jclinpath-2017-204524 28821583

[32] Urrechaga E Bóveda O Aguirre U. Improvement in detecting sepsis using leukocyte cell population data (CPD). Clin Chem Lab Med. (2019) 57:918–26. 10.1515/cclm-2018-0979 30838839

[33] Buoro S Seghezzi M Vavassori M Dominoni P Esposito SA Manenti B. Clinical significance of cell population data (CPD) on Sysmex XN-9000 in septic patients with or without liver impairment. Ann Transl Med. (2016) 4:418. 10.21037/atm.2016.10.73 27942509 PMC5124619

[34] Shalini P Purnima S. Rao, Sneha Rao AR, Manjula Anil, Athira Benny, Sandhya I. Diagnostic utility of cell population data (cpd) in sepsis using automated hematology analysers. Ann Pathol Lab Med. (2019) 6:A284–8. 10.21276/apalm.2395

[35] Urrechaga E. Reviewing the value of leukocytes cell population data (CPD) in the management of sepsis. Ann Transl Med. (2020) 8:953. 10.21037/atm-193173 32953753 PMC7475430

[36] Crouser ED Parrillo JE Seymour C Derek C Angus Keri Bicking Vincent G Esguerra. Monocyte distribution width, a novel indicator of Sepsis-2 and Sepsis-3 in high risk emergency department patients. Crit Care Med. (2019) 47:1018–25. 10.1097/CCM.0000000000003799 31107278 PMC6629174

[37] Giannoni E Schlapbach LJ. Editorial: sepsis in neonates and children. Front. Pediatr. (2020) 8:621663. 10.3389/fped.2020.621663 33330295 PMC7728741

[38] Singer M Deutschman CS Seymour CW Shankar-Hari M Annane D Bauer M. The third international consensus definitions for sepsis and septic shock (Sepsis-3). JAMA. (2016) 315:801–10. 10.1001/jama.2016.0287 26903338 PMC4968574

[39] Segal AW. How neutrophils kill microbes. Annu Rev Immunol. (2005) 23:197–223. 10.1146/annurev.immunol.23.021704.115653 15771570 PMC2092448

[40] Silvestre-Roig C Hidalgo A Soehnlein O. Neutrophil heterogeneity: implications for homeostasis and pathogenesis. Blood. (2016) 127:2173–81. 10.1182/blood-2016-01-688887 27002116

[41] Yona S Jung S. Monocytes: subsets, origins, fates and functions. Curr Opin Hematol. (2010) 17:53–9. 10.1097/MOH.0b013e3283324f80 19770654

[42] Raimondi F Ferrara T Capasso L Sellitto M Landolfo F Romano A. Automated determination of neutrophil volume as screening test for late-onset sepsis in very low birth infants. Pediatr Infect Dis J. (2010) 29:288. 10.1097/INF.0b013e3181c37fb4 20190619

[43] Jung YJ Kim JH Park YJ Kahng J Lee H Lee KY. Evaluation of cell population data on the UniCel DxH 800 Coulter Cellular Analysis system as a screening for viral infection in children. Int J Lab Hematol. (2012) 34:283–9. 10.1111/j.1751-553X.2011.01392.x 22226427

