# Supplementary material for: Cell Population Data (CPD) for Early Recognition of Sepsis and Septic Shock in Children: A Pilot Study
Source: Front Pediatr. 2021 Mar 8;9:642377. doi: 10.3389/fped.2021.642377 (PMC7989813; doi:10.3389/fped.2021.642377)
Supplement: Supplementary file 1 [file Data_Sheet_1.PDF]

**Table 1S**. Cell Population Data (CPD) parameters and morphological and functional characteristics of leukocytes.

| CPD parameters | Morphological and functional characteristics of leukocytes |
|---|---|
| **NE-SFL**<br>(neutrophils fluorescence intensity) | It reflects the amount of cellular DNA and RNA, such as in immature granulocytes, band neutrophil |
| **MO-X**<br>(monocytes cells complexity) | It reflects amounts of granules, vacuoles and other cytoplasmic inclusions |
| **MO-Y**<br>(monocytes fluorescence intensity) | It reflects the amount of cellular DNA and RNA |
| **MO-WX**<br>(monocytes complexity and width of dispersion of the events measured) | It reflects the degree of heterogeneity of monocytes population, compared to MO-X |
| **MO-WZ**<br>(monocytes cells size and width dispersion) | It reflects the degree of heterogeneity of the monocytes population, compared to MO-Z |
