# Supplementary material for: Cell Population Data (CPD) for Early Recognition of Sepsis and Septic Shock in Children: A Pilot Study
Source: Front Pediatr. 2021 Mar 8;9:642377. doi: 10.3389/fped.2021.642377 (PMC7989813; doi:10.3389/fped.2021.642377)
Supplement: Supplementary file 2 [file Data_Sheet_2.PDF]

Table 2S. CPD values upon PICU admission *(t0)* in subgroups of patients with sepsis, septic shock and control group. Data expressed as median and interquartile range (IQR). (* p<0,01) between G1a and G2; ** p<0,01) between G1b and G2)

**CPD at *t0* median (IQR)**	**G1a - Sepsis subgroup (n=20)**	**G1b - Septic shock subgroup (n=6)**	**G2 - Control group (n=40)**
NE-SFL	**51,7 *** (49,7-57,7)	**59,9 **** (54,3-65)	**46,8** (45,9-47,8)
MO-X	**121,3** (118,4-124,5)	**126,3 **** (118,6-130,4)	**121** (119,7-122,1)
MO-Y	**109,9** (105,4-121,9)	**111,8 **** (104,3-138,5)	**108,1** (102,8-111,3)
MO-WX	**274 *** (244,5-300,2)	**272 **** (260,5-338,2)	**249,5** (231-269)
MO-WZ	**587** (537-597,5)	**516** (487,7-591,5)	**572,5** (526,5-629,5)
