# Supplementary material for: Cell Population Data (CPD) for Early Recognition of Sepsis and Septic Shock in Children: A Pilot Study
Source: Front Pediatr. 2021 Mar 8;9:642377. doi: 10.3389/fped.2021.642377 (PMC7989813; doi:10.3389/fped.2021.642377)
Supplement: Supplementary file 3 [file Data_Sheet_3.PDF]

Table 3S. CPD values upon PICU admission *(t0)* in patients with uncomplicated (G1a subgroup) and complicated sepsis course (G1b subgroup). Sepsis course was defined as complicated if patients either had severe sepsis or died for sepsis-induced multiple organ failure. Data expressed as median and interquartile range (IQR).

**CPD at *t0* median (IQR)**	**G1a – Uncomplicated sepsis course (n=16)**	**G1b – Complicated sepsis course (n=10)**	*p value*
NE-SFL	**52,5**<br>(50,1-56,6)	**55,7**<br>(49,3-65)	0,26
MO-X	**121**<br>(117,4-122,9)	**124,8**<br>(119,2-129,1)	0,1
MO-Y	**107,3**<br>(100,1-121,3)	**114,7**<br>(107,9-130,1)	0,11
MO-WX	**282**<br>(257,5-300,2)	**257,5**<br>(248-316,7)	0,88
MO-WZ	**587**<br>(525,5-597,5)	**548**<br>(518-616)	0,96
