# Supplementary figures and images for: Cell Population Data (CPD) for Early Recognition of Sepsis and Septic Shock in Children: A Pilot Study
Source: Front Pediatr. 2021 Mar 8;9:642377. doi: 10.3389/fped.2021.642377 (PMC7989813; doi:10.3389/fped.2021.642377)

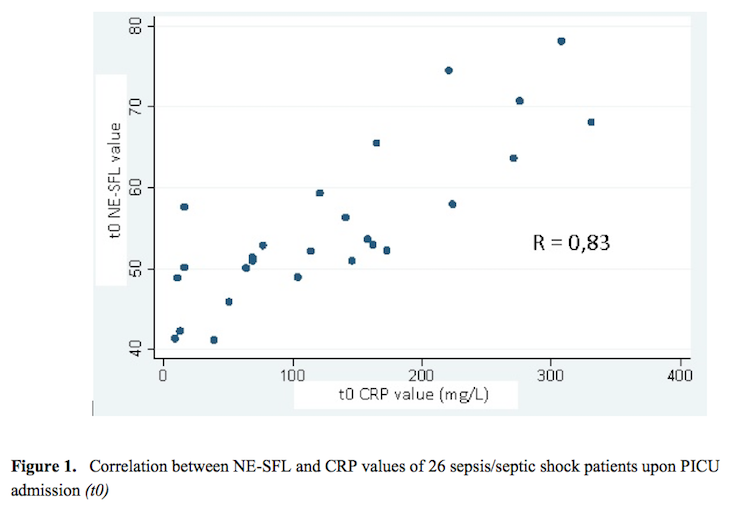

Supplement: Supplementary file 4 [file Image_1.TIFF]
